# Assessing the Effect of Climate Variables on the Incidence of Dengue Cases in the Metropolitan Region of Panama City

**DOI:** 10.3390/ijerph182212108

**Published:** 2021-11-18

**Authors:** Vicente Navarro Valencia, Yamilka Díaz, Juan Miguel Pascale, Maciej F. Boni, Javier E. Sanchez-Galan

**Affiliations:** 1 Facultad de Ciencias y Tecnología, Universidad Tecnológica de Panamá (UTP), El Dorado 0819-07289, Panama; vicente.navarro@utp.ac.pa; 2 Department of Research in Virology and Biotechnology, Gorgas Memorial Institute of Health Studies, Justo Arosemena Avenue and 35st Street, Panama 0816-02593, Panama; ydiaz@gorgas.gob.pa; 3 Unit of Diagnosis, Clinical Research and Tropical Medicine, Gorgas Memorial Institute of Health Studies, Justo Arosemena Avenue and 35st Street, Panama 0816-02593, Panama; jmpascale@gorgas.gob.pa; 4 Sistema Nacional de Investigación (SNI) SENACYT, Panama 0816-02852, Panama; 5 Center for Infectious Disease Dynamics, Department of Biology, Pennsylvania State University, University Park, PA 16802, USA; mfb9@psu.edu; 6 Grupo de Investigaciones en Biotecnología, Bioinformática y Biología de Sistemas (GIBBS), Facultad de Ingenieria de Sistemas Computacionales, Universidad Tecnológica de Panamá (UTP), El Dorado 0819-07289, Panama

**Keywords:** dengue, time series, climatic variables, correlation, prediction, SARIMA, SARIMAX, RNN, LSTM

## Abstract

The present analysis uses the data of confirmed incidence of dengue cases in the metropolitan region of Panama from 1999 to 2017 and climatic variables (air temperature, precipitation, and relative humidity) during the same period to determine if there exists a correlation between these variables. In addition, we compare the predictive performance of two regression models (SARIMA, SARIMAX) and a recurrent neural network model (RNN-LSTM) on the dengue incidence series. For this data from 1999–2014 was used for training and the three subsequent years of incidence 2015–2017 were used for prediction. The results show a correlation coefficient between the climatic variables and the incidence of dengue were low but statistical significant. The RMSE and MAPE obtained for the SARIMAX and RNN-LSTM models were 25.76, 108.44 and 26.16, 59.68, which suggest that any of these models can be used to predict new outbreaks. Although, it can be said that there is a limited role of climatic variables in the outputs the models. The value of this work is that it helps understand the behaviour of cases in a tropical setting as is the Metropolitan Region of Panama City, and provides the basis needed for a much needed early alert system for the region.

## 1. Introduction

As the global population grows and becomes more connected, human pathogens that were previously localized may expand their range and require greater collective action for disease control. One of the more likely candidates for geographic expansion is dengue virus (DENV), an *Aedes*-transmitted flavivirus that infects hundreds of millions of people per year [1] and is predicted to expand its range as the climate warms [2]. DENV circulates in the global tropics as four distinct serotypes (called DENV1, DENV2, DENV3 and DENV4) that show seasonal epidemic patterns, endemic behavior, and infrequent outbreaks, depending on the regional climate and altitude. Dengue hospitalizations number in the single-digit millions per year globally [3,4] presenting both a health and an economic burden [4,5]. After reintroduction of DENV to the Americas in 1980, the first endemic case of dengue was recorded in Panamanian territory in 1993 [6]. Since then, all four serotypes of DENV have been circulating in the canal lands for more than 25 years [7].

Proximity to breeding grounds of either *Aedes aegypti* and *Aedes albopictus* mosquitoes enhances the risk of transmission of the virus from one host to another [8]. It is known that typically an increase number of mosquitoes is reflected in an relative increase of the dengue cases and in Panama as in most tropical places, this increase occurs in the rainy season [9]. Moreover, dengue cases in Panama have been studied as spacial-temporal clusters of larvae and incidence [10,11], have been related to the existence and prevalence of used tires [12], and even cases have been related to socio-economical status [13]. However, to the best of our knowledge, there is no study relating climatic conditions with the incidence of dengue cases in the Metropolitan region of Panama.

### Modeling the Dengue Incidence as a Time Series

Correlation, modeling, and prediction methods have been applied extensively to understand epidemics such as dengue. Most studies focus on the human host, accounting for the confirmed cases and from there trying to understand its relationship with climatic variables [14]. Other focus on the vector proliferation in all its stages in order to understand the dynamic of the vector incidences and its relation to cases incidence [15].

A wealth of studies trying to model Dengue incidence use statistical techniques, borrowed from time series analysis, to be able to model and predict new events of the series. This studies often make use of univariate methods, as the Autoregressive Integrated Moving Average-ARIMA, which tries to explain the values of the event of the series based on its own lags, moving averages and autoregressive components [16,17].

Other studies make use of multivariate methods, that are able to relate the incidence series to other variables that might be influential to the series. In that case, seasonal effects are taking into consideration, for instance: natural climate variations that happen during the year and the change of seasons. Some of the methods under this category are: Seasonal Autoregressive Integrated Moving Average-SARIMA and Seasonal Autoregressive Integrated Moving Average with eXogenous factors-SARIMAX [18,19,20,21,22].

More recently, studies have started to use machine learning techniques (neural networks and deep neural networks) focused on time-series analysis for studying this type of relationships [23].

For instance, recurrent neural networks (RNN) with long-short-term memory (LSTM) type, have been applied to forecast the incidence in 20 Chinese cities from climatic variables and historical cases [24]. Furthermore, hybrid methods using both traditional statistical and LSTM have been studied, evaluating their ability to reproduce epidemic events seen for dengue, as well as predict future incidence [25]. Also, implementations of stacking models have been made, where stacking involves averaging predictions of multiple models using a weighted average, these models outperform the Bayesian moving average [26]. Finally, social networks have been used be a potential predictor in forecasting the number of reported cases [27].

The objectives of this study are as follows: (1) assess the relation between dengue incidence cases and its relation with rural or urban settings in the metropolitan region of Panama; (2) to assess the correlation between confirmed dengue cases and air temperature, precipitation and relative humidity in the metropolitan region of Panama; (3) Assess if time series regression based models (SARIMA and SARIMAX) or a recursive neural network based model (RNN-LSTM) can learn the incidence pattern and able to predict the future behaviour of the series.

## 2. Materials and Methods

### 2.1. Dengue Incidence Data

Dengue incidence data were compiled by the Gorgas Memorial Institute of Health Studies (ICGES) and the Ministry of Health (MINSA). The data collection program was founded by the MINSA in 1988, and the data used in the present analysis is a subset of that described in Diaz et al. [7]. The definition of a dengue case follows the guidelines of the World Health Organization (WHO). From 1993 to 2011, the 1997 classification of clinical cases of Dengue fever (DF), Dengue with hemorrhagic fever (DHF), and Dengue shock syndrome (DSS) was applied. From 2012 forward, the definition of dengue with symptoms or without symptoms and severe dengue provided by WHO 2009 was applied [28]. ICGES, located in Panama City, was the primary site for conducting DENV laboratory assays for inpatients and outpatients in provincial hospitals in Panama who had fever that was clinically suspected to be dengue.

**Figure 1:**
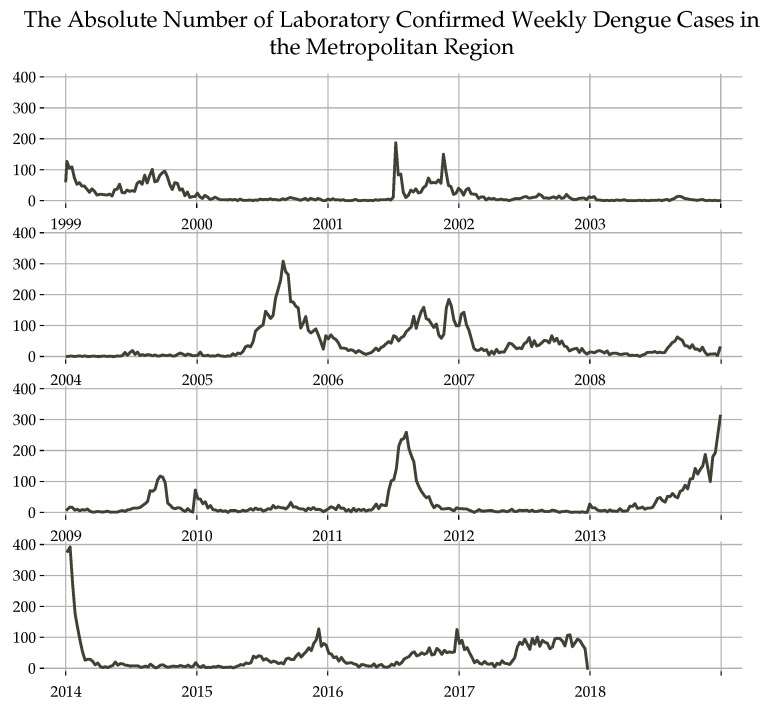
Map with the scale distribution of the accumulated number of reported cases of Dengue in the Metropolitan Region.

The data were aggregated by epidemic week and include all laboratory-confirmed dengue cases from January 1999 to December 2017, as it is shown in Figure 1.

**Figure 2:**
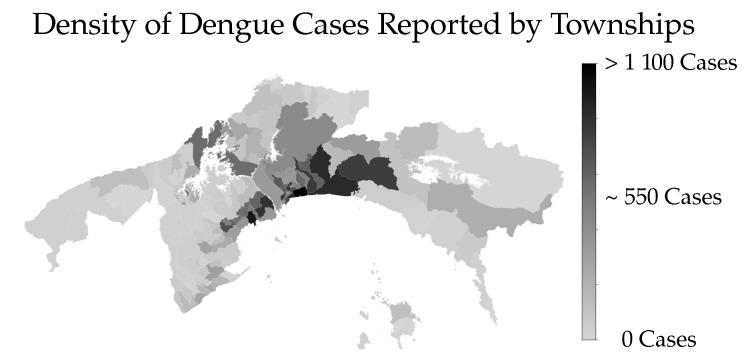
Map with the scale distribution of the accumulated number of reported cases of Dengue in the Metropolitan Region.

The Metropolitan region, comprises the regions with the highest population density of the country, with more than 1.5 millions inhabitants. It comprises the provinces of Panamá, Panamá Oeste and Colón. It has with an area of approximately 14,034 km${}^{2}$ and an average elevation of 80 (meters above sea level-MASL) [29]. Figure 2 shows the distribution by township of the accumulated confirmed cases of Dengue.

Many authors mention that the direct factors in the transmission of Dengue are both the climatic variables and the population density [12,13,30]. In order to assess the second premise about population density the national definition of rural areas, basically speaking, areas formed by the group of townships with less than 1500 inhabitants [29]. The whole metropolitan areas and the number of cases per-capita (cases per thousand people) in rural vs. urban areas were compared.

### 2.2. Climate Data

**Figure 3:**
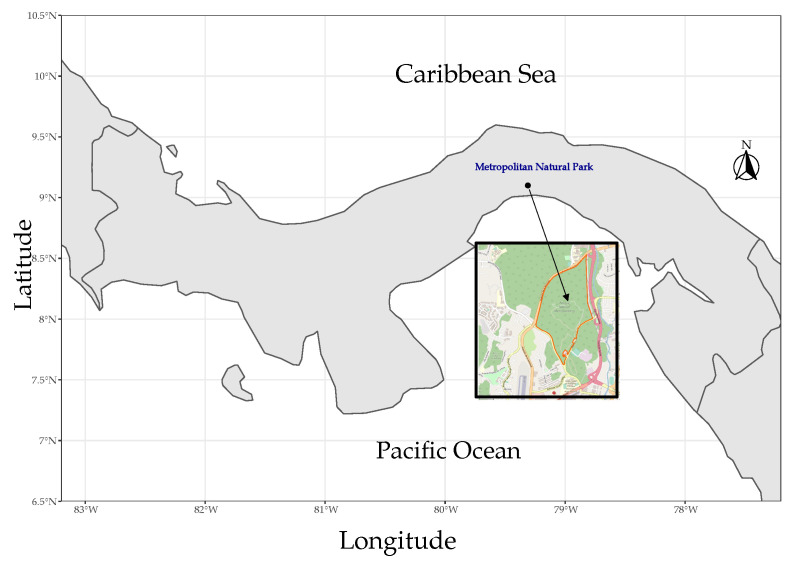
Map with the location of the Metropolitan Natural Park.

The data set of climatic variables were taken from the Metropolitan Park meteorological station (8.9944, −79.5430), belonging to the network of The Smithsonian Tropical Research Institute (STRI) [31,32,33]. The station is located in the extreme northwest of Panama City, at the eastern end of the Metropolitan Natural Park. It is surrounded by an 80-years-old semi-deciduous lowland forest. Figure 3 shows the exact location of the station, in relation to Panama City and the rest of the Country.

The full data set covers climate variables from 1995 to present, but it was arranged to cover period as dengue cases incidence. This weather station was chosen because it had an elevation similar to the average elevation of the studied area of about 94 MASL.

Three time series of interest were selected from this station: air temperature, precipitation and relative humidity. Empty data points were imputed using a multivariate deep learning LSTM model and the nearby station of Barro Colorado, similar to the procedure described in [34].

### 2.3. Time Series Creation

The confirmed cases were indexed by epidemiological week as a time series. The same was done for the climatic variables. However, for the air temperature and relative humidity the weekly average was used. As for precipitation, the accumulated amount of rain during the week was used.

Having all four series at the same interval (Equation (1)), was the first step to a series of analyses that will be explained in the next subsections.

(1) $$Y_{i}=Y_{i,1},Y_{i,2},Y_{i,3},\ldots ,Y_{i,t}:t\in T;\left|T\right|=991\;\mathrm{weeks}$$

$$\mathrm{where}\;i\;\mathrm{is}\;\mathrm{the}\;\mathrm{time}\;\mathrm{series}\;=\left\{\begin{array}{cc} 1, & \mathrm{dengue}\;\mathrm{incidence}\\ 2, & \mathrm{air}\;\mathrm{temperature}\\ 3, & \mathrm{relative}\;\mathrm{humidity}\\ 4, & \mathrm{precipitation}\;\left(\mathrm{rain}\right)\; \end{array}\right.$$

#### 2.3.1. Correlation

Once all time series were created, the second objective was to define if there was a correlation among them. For this matter, both the Pearson correlation coefficients (Equation (2)) [35] and the Spearman’s rank correlation coefficient (Equation (3)) [36], were calculated.

(2) $$\rho_{Y_{i},Y_{j}}=\frac{cov(Y_{i},Y_{j})}{\sigma_{Y_{i}}\sigma_{Y_{j}}}$$

(3) $$\rho_{R\left(Y_{i}\right),R\left(Y_{j}\right)}=\frac{cov(R\left(Y_{i}\right),R\left(Y_{j}\right))}{\sigma_{R(Y_{i})}\sigma_{R(Y_{j})}}$$

where cov(·) is the represents the covariance calculation between two time series, $\sigma_{\cdot }$ is the standard deviation of the series and R(·) are the series values converted to ranks.

#### 2.3.2. Prediction

Resulting significant correlations were used to predict the Dengue incidence time series $Y_{1,t}$. Three different types of models *M* capable of learning the parameters of this time series and predicting ${\hat{y}}_{1,t}=M\left(x_{1},x_{2},\ldots ,x_{T}\right)$ were used.

The first two models being of the Moving Average Models (MA) family and the last one being a recurrent neural network method:

1. *SARIMA*: these models use non-seasonal differences, auto-regressions and moving average data from previous samples, and seasonal differences, auto-regressions and moving average from previous periods, which allows them to accurately predict the next steps of a time series, with the assumption that the same behavior is maintained in the causes of these events.
  The mathematical formulation of SARIMA models can be generalized as described in (Equation (4)).
  (4) $$\Delta_{S}^{D}\Delta^{d}Y_{t}=\frac{\Theta_{Q}\left(B^{S}\right)\theta^{q}\left(B\right)\epsilon_{t}}{\Phi_{P}\left(B^{S}\right)\phi^{p}\left(B\right)}$$
  In this equation *p*, *d* and *q* represent the non-seasonal order of Auto-Regression (AR), differentiation and Moving Average (MA, respectively. P, D, and Q represent the seasonal order of AR, differentiation, and MA respectively. Moreover, $Y_{t}$ represents the time series data in period *t*. $\epsilon_{t}$ represents the Gaussian white noise process (random walk) in period *t*. *B* represent the backward shift operator ($B^{k}x_{t}=x_{t-k}$). $\Delta_{S}^{D}$ represents the seasonal difference. $\Delta^{d}$ represents the non-seasonal difference. *S* represent the seasonal order (S=52 for weekly data analysed yearly).
2. *SARIMAX*: these models are related to SARIMA and share the same variables of the model, however they include exogenous or explanatory variables of the time series. Also, they can be formulated adding a vector or matrix that represents the exogenous variables and his respective weights that represents the influence or contribution of these variables to the regression, as it can be seen in (Equation (5)):
  (5) $$\Delta_{S}^{D}\Delta^{d}Y_{t}=\frac{\Theta_{Q}\left(B^{S}\right)\theta^{q}\left(B\right)\epsilon_{t}+\beta_{k}x_{k,t}}{\Phi_{P}\left(B^{S}\right)\phi^{p}\left(B\right)}$$
  In SARIMAX models, $x_{k,t}$ represents the vector or matrix that includes the k-th exogenous variable. $\beta_{k}$ represents the coefficient or weight that accompanies the k-th exogenous variable, all the coefficients are adjusted to the data in order to obtain the value that best relates their influences to the time series. Models that use seasonal variables are also called models with long-term memory.
3. *RNN-LSTM*: Recurrent Neural Networks (RNN), as with any neural network, can be used to adjust a non-linear model, by learning long-term dependencies that could be present in a data set, thus can be used to describe and model a time series. Although in practice what is known as an explosion or disappearance of the gradient happens [37]. A solution to this technical problem, is using networks with long-short-term memory (LSTM) [38] that in general perform better than RNNs.
  The advantage of LSTM networks lies in the way the hidden state is computed, in the iteration *t*, the output state (see Equation (11)), is calculated using the result of four components as input, known as follows: input gate (Equation (6)), forget gate (Equation (7)), output gate (Equation (8)) and a cell state (Equations (9) and (10)).
  Gates:
  (6) $$i_{g}=\sigma \left(R_{ia}h_{t-1}+W_{ix}x_{t}+b_{i}\right)$$
  (7) $$f_{g}=\sigma \left(R_{fa}h_{t-1}+W_{fx}x_{t}+b_{f}\right)$$
  (8) $$o_{g}=\sigma \left(R_{oa}h_{t-1}+W_{ox}x_{t}+b_{o}\right)$$
  where σ is a nonlinear activation function, for example, the sigmoid function, $R_{ix}$ and $W_{ia}$ are the recurring and input weights respectively, $b_{i}$ is the activation threshold or bias.
  Cell state:
  (9) $$g_{t}=\tanh\left(R_{ga}h_{t-1}+W_{gx}x_{t}+b_{g}\right)$$
  (10) $$C_{t}=f_{g}⊙C_{t-1}+i_{g}⊙g_{t}$$
  Hidden state:
  (11) $$h_{t}=o_{g}⊙\tanh\left(C_{t}\right)$$
  ⊙ represents the Hadamard’s product.

**Figure 4:**
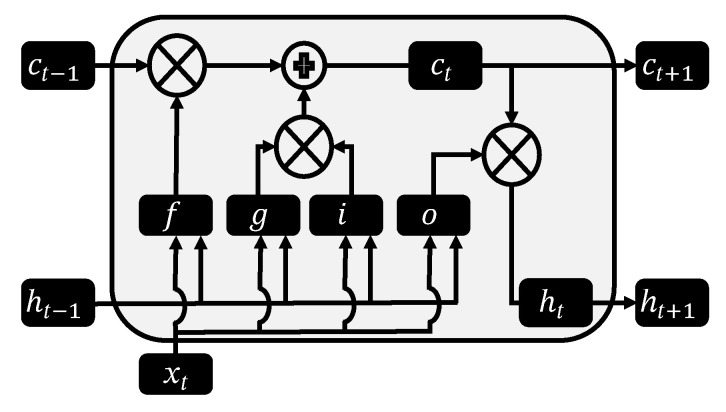
Graphical representation of an RNN of LSTM type.

Figure 4 provides a visual representation on how this gates and cells interact inside each unit (neuron) of the recurrent neural network.

The architecture of the LSTM network used for this task, had an input layer, an LSTM Layer, a dropout Layer, a second LSTM Layer, a dropout Layer, and a final regression layer at the output. The dropout layers in the training process have and input parameter of 0.45, a learning rate of 5 × 10${}^{-6}$ was used, mini-batch size of 204 values (4 years of data were used), the LSTM have the same amount of hidden units (H.U.).

Once the network was trained the learned weights approximate/predict the following weekly values by actualizing the inner state with all the previous data an then using 12 weeks of data to predict the next one successively.

The features used to predict the Dengue incidence in the step *t* were the incidence, air temperature, precipitation and relative humidity of the step t−1. The in-sample prediction take the past true value of the Dengue Incidence to predict the future one, the out-sample prediction take the past prediction to predict the next one successively.

#### 2.3.3. Error Metrics

All three models were compared under the Root Mean Square Error (RMSE) (Equation (12)) and Mean Absolute Percentage Error (MAPE) (Equation (13)) performance metrics [37].

(12) $$\mathrm{RMSE}=\sqrt{\frac{1}{n}\sum_{i=1}^{n}{\left({\hat{y}}_{1,t}-Y_{1,t}\right)}^{2}}$$

(13) $$\mathrm{MAPE}=\frac{100}{n}\sum_{i=1}^{n}\frac{\left|{\hat{y}}_{1,t}-Y_{1,t}\right|}{\left|Y_{1,t}\right|}$$

The RMSE metric is a way of measuring how much a model fits to a set of data, and it can be a regression or prediction model as in our case, and MAPE measures the cumulative percentage error of a data set generated by a model on another set of target data.

#### 2.3.4. Data Pre-Processing and Software

The data set was normalized within the [0,1] interval. In total 16 years (1999–2014) of data were used to train the models, and performance metrics were compared by predicting 3 years. For the organization of the data a spreadsheet software (Apache OpenOffice Calc 4.1.11, Apache Software Foundation) was used. For all the visualizations, correlation analysis and regression models (SARIMA and SARIMAX), the R statistical software was used, in specific the forecast, stats, xts packages. The RNN-LSTM network was implemented using the Deep-Learning Toolbox of Matlab 2021a.

## 3. Results

### 3.1. Urban vs. Rural by Population Density in the Metropolitan Region

**Figure 5:**
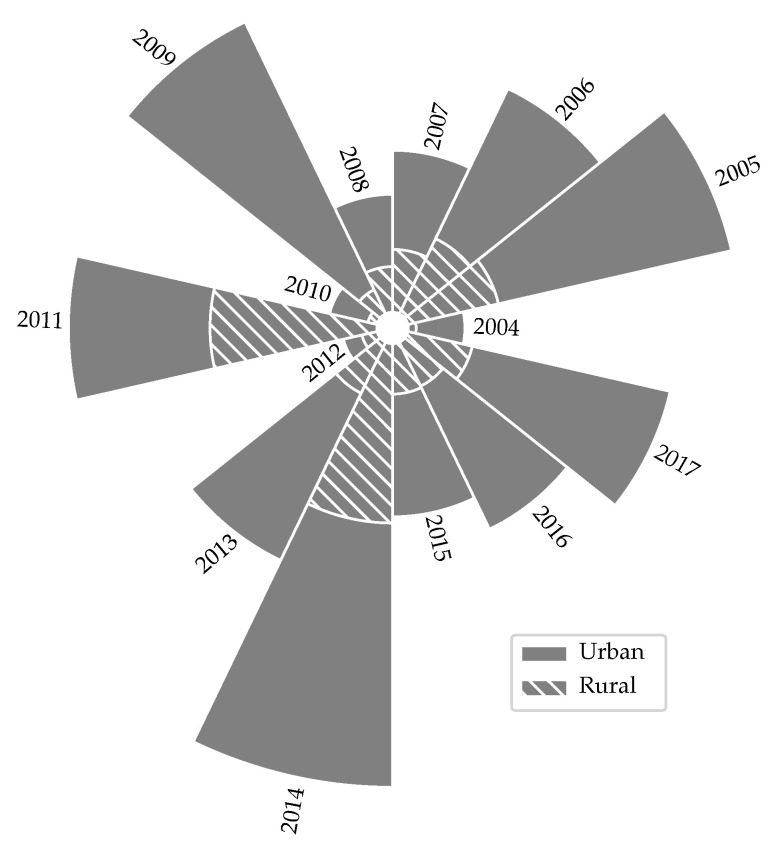
Portion of the number of cases per-capita in urban and rural areas per year.

As the first objective of this work was to assess the relation between dengue incidence cases and its relation with population density. First the incidence was tabulated per township size, using all data collected on dengue cases, including laboratory confirmed and epidemiological link (suspicious) cases. Also, the cumulative number cases, were aggregated distinguishing between rural and urban areas metropolitan region of Panama. Figure 5 shows the numbers of cases in rural and urban areas per 1000 cases in the 2004–2017 (unregistered and/or missing values caused to drop the 1999–2003 period). Furthermore, a significant difference was confirmed via a T-test (*p* < 0.01) when comparing both areas per year, see Appendix A. Confirming the relationship between population density and cases for the metropolitan region of Panama.

The data presented in Figure 5 shows a relationship between population density and the number of cases per-capita. This information have been used by local authorities to direct efforts to control Dengue outbreaks. The main strategies include the elimination of hatcheries and the eradication of vectors. Although, the same does not happen in less populated areas, where the virus is circulating almost freely. This is important to the light of recent studies by Whiteman et al. [13] and Bennett et al. [12] which state that in the not so populated areas the vector Aedes Albopictus is more active and in the more densely populated areas it is the Aedes Aegypti the most active vector.

### 3.2. Preliminary Series Analysis

**Figure 6:**
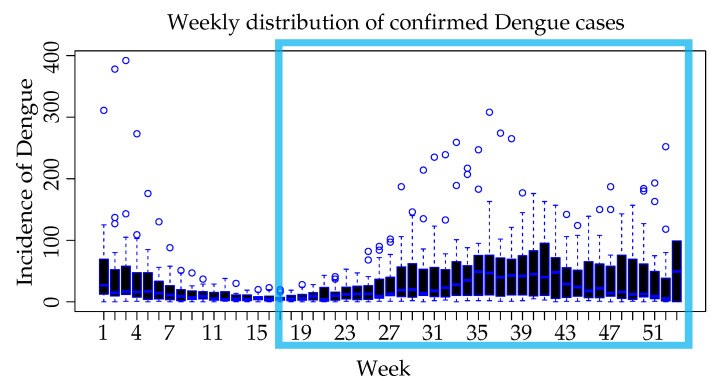
Weekly distribution of confirmed dengue cases, grouped by weeks along the study years.

This subsection compiles the preliminary statistical analysis carried out on the Dengue incidence and the climatic variables. The data throughout the study were grouped by week in order to better understand the influence of climatic variables and how they change over the year. As it can be seen in Figure 6, the distributions of confirmed Dengue cases over the years allows us to see that approximately 7–8 weeks after the onset of rain season (highlighted with light blue square) the incidence of Dengue cases tends to increase.

The intervals comprised by weeks 1–3 and 33–42 registered the highest average number of cases (>48 cases per week), with a value greater than the third quartile of the averages of all the weeks. In weeks 10–22, the lowest average number of cases was recorded (<12 cases per week), corresponding to a value lower than the first quartile. The highest number of cases registered in one week was 392.

The results of the incidence of Dengue, are directly influenced by the actions taken to prevent infections, public health strategies have demonstrated a high effectiveness in the control of vectors and with it in the control of Dengue outbreaks. However, there is a relationship with the climatic variables that could shed light on the critical moments that could improve the effectiveness of these strategies, then the climatic variables will be analyzed statistically to seek to understand the key moments in the prevention of Dengue epidemics.

**Figure 7:**
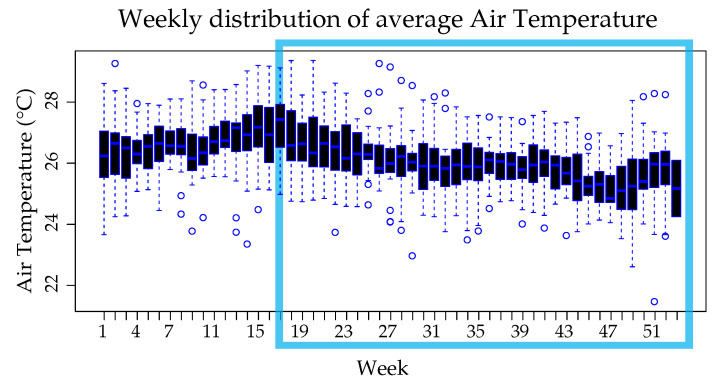
Weekly average air temperature distribution, the weekly average air temperature was grouped by week to visualize changes over the years by week.

Figure 7 shows that the highest average air temperatures (>26.3 ${}^{\circ }$C) were recorded in the weeks interval of 7–21 corresponding to the values above the third quartile, from weeks 42–51 the lowest average temperatures (<25.6 ${}^{\circ }$C) were recorded. This fits in with the sunniest months of summer and the rainiest months of winter respectively.

**Figure 8:**
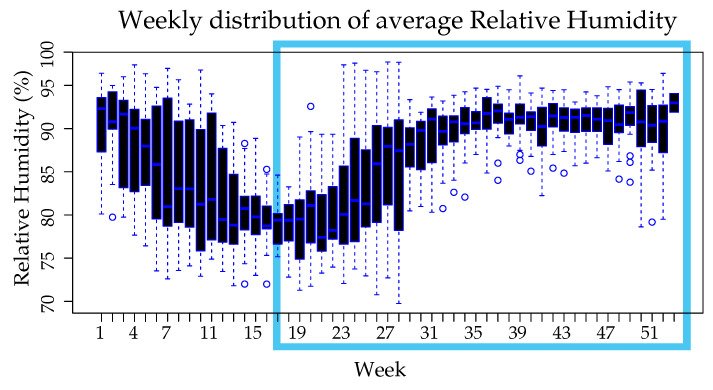
Weekly distribution of average Relative Humidity, grouped by week along the study period.

Figure 8 shows that from week 11–22 the lowest values of average relative humidity (<82%) were recorded, weeks 1–2 and 34–52 present the highest average relative humidity (>90%) with values higher than the third quartile, this includes the rainiest months (mid-September-October-November-mid-December) and it can be seen that even after the rains are over, the environment remains humid until about 4 weeks later. Panama tends to be a region with a high relative humidity, even more so in the summer season it is possible to find itself dry enough to influence the decrease in the breeding grounds of mosquitoes.

**Figure 9:**
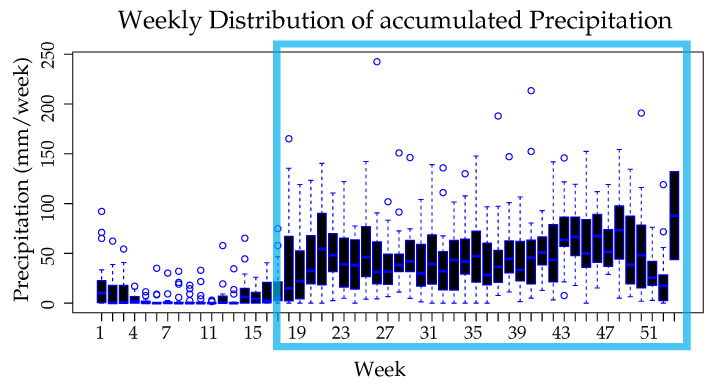
Weekly distribution of accumulated Precipitation, grouped by week along the study period.

As it can be seen in Figure 9, from week 4 to week 13 the lowest amount of accumulated precipitation was recorded (<11 mm/week), this correspond to the lowest part of the first quartile. Weeks 38–50 register the highest amount of accumulated precipitation (>52 mm/week) as part of the upper third quartile. The beginning of the rainy season marks a turning point where the average temperature begins to decrease and the average relative humidity to increase.

### 3.3. Correlation Analysis

**Figure 10:**
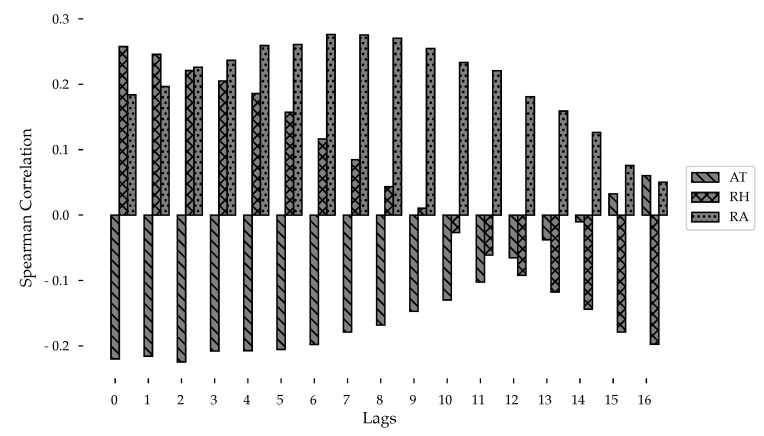
Variation in Spearman’s correlation coefficient as climatic variables are lagged.

Results confirm that there is a correlation between the climatic variables and the incidence of Dengue. Both correlation coefficients (Spearman’s and Pearson’s) provide a measure of how strong these relationships are in different moments or lags. The summary of the correlation coefficients using the Spearman method are shown in Figure 10. Where air temperature is refered as AT, relative humidity as RH, and precipitation as RA.

The monotonic correlation has higher values and which vary more abruptly, while the linear correlation is smaller and varies smoothly. The correlation analysis supports what has been found statistically about the relationship in an increase in the incidence of Dengue with the climatic variables, it gives us light on the time in which these variables are reflected in the incidence of Dengue, being approximately at the time of the highest correlation (8–9 weeks before). The most significant correlations (*p* < 0.001) are those with a coefficient greater than 0.11.

**Figure 11:**
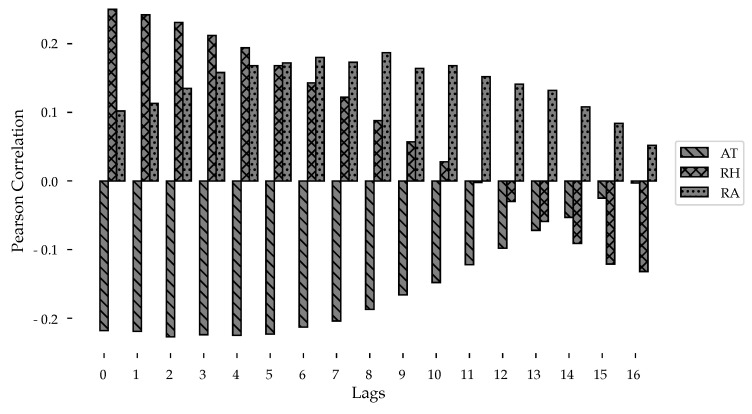
Variation in Pearson’s correlation coefficient as climatic variables are lagged.

Figure 11 shows the correlation calculated by the Pearson coefficient and its lags.

In this case, Air Temperature shows its strongest relationship with Dengue incidence two weeks later, Precipitation has its highest correlation at a lag of 8 weeks, and Relative Humidity maintains it’s stronger correlation without lag. The most significant correlations (*p* < 0.001) are those with a correlation coefficient greater than 0.10.

### 3.4. Prediction

The three different models were assessed with the variables and lags extracted from the correlation analysis described in the previous section.

**Figure 12:**
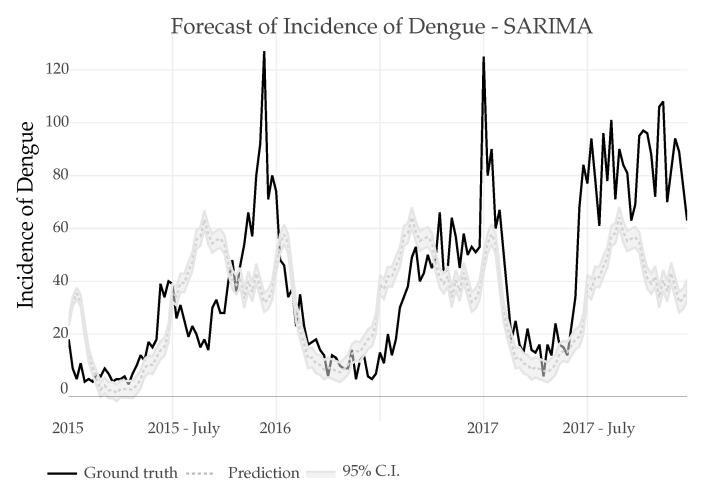
Prediction of Dengue Incidence for 3 years using a SARIMA model and comparison with the real number of cases.

**Figure 13:**
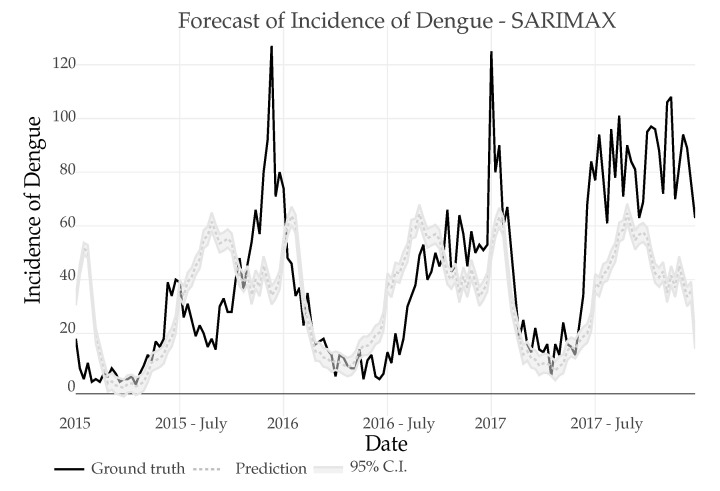
Prediction of Dengue Incidence for 3 years using a SARIMAX model and comparison with the real number of cases.

1. *SARIMA*: The predictions were made using 16 years of training data (835 weeks) to predict the future index in the next 3 years (156 weeks), seasonality of the series was achieved after a (1) difference, in Figure 12 the prediction can be seen, the minimum amount of data necessary to make the prediction was also tested where a minimum of 6 years of data were needed to maintain a similar error metric to the one shown.
2. *SARIMAX*: The SARIMAX model uses the data of the Dengue incidence, the Air temperature with a lag of 2, the precipitation with a lag of 8 and the relative humidity with a lag if 0. As it can be seen in Figure 13 the results are very similar to those obtained using SARIMA.

In both cases, regression models for SARIMA and SARIMAX make a prediction error, presenting a peak just at the beginning of the predicted series. This could be attributed to the presence of a heavy periodic temporal component and the existence of a similar peak just in the previous period (see 2014 peek in Figure 1).

3. *LSTM*: In the prediction using RNN-LSTM, an initial configuration of parameters that best adjusted to the training set was first tested. Then, it was verified by making a prediction within the sample set (using the real Dengue incidence to predict the future incidence in each step).

**Figure 14:**
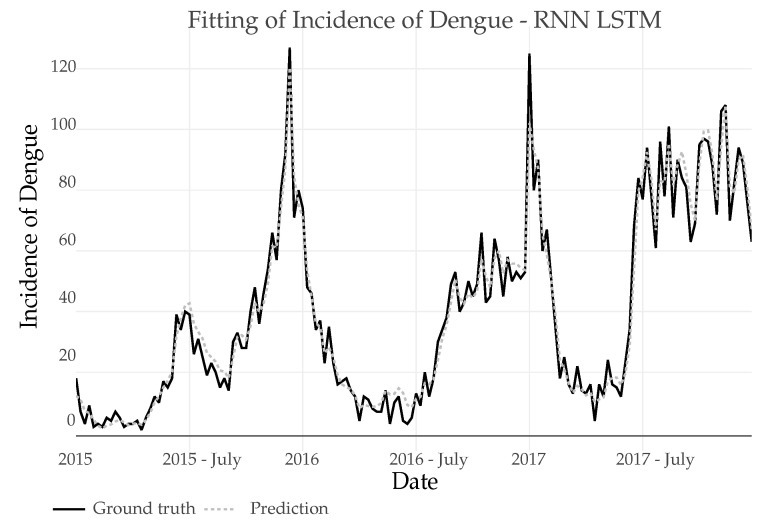
Fitting of Dengue Incidence for 3 years using a RNN-LSTM in-sample model and comparison with the real number of cases.

Figure 14 shows the seamlessly fit (shown in dotted line pattern) of the RNN-LSTM model tested by making a prediction within the sample.

**Figure 15:**
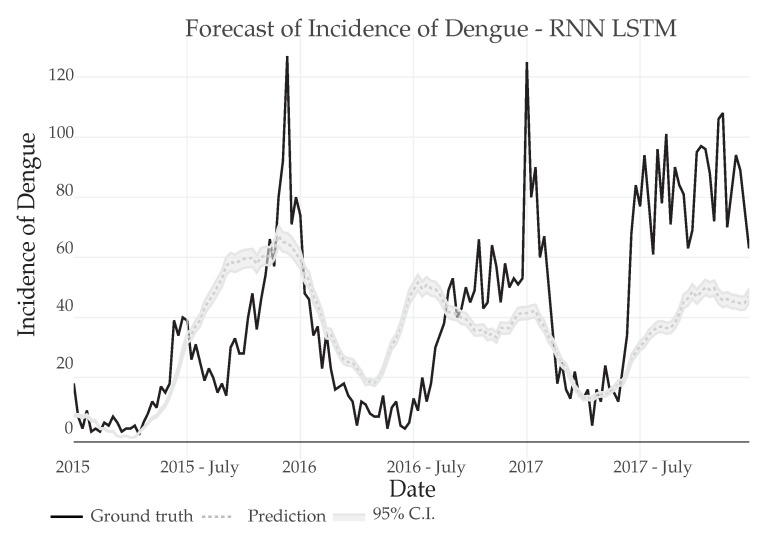
Prediction of Dengue Incidence for 3 years using a RNN-LSTM out-sample model and comparison with the real number of cases.

After the adjustment and verification of the RNN preliminary results, the prediction was carried out outside the sample set, using only the training data to successively generate each point of the future Dengue incidence, as it can be seen in Figure 15.

It is of importance to notice, that even when out-of-sample prediction were made it still is able to predict a general pattern of the incidence series, with predicted peaks close to every elevation of cases in the 2015–2017 period.

### 3.5. Model Evaluation

The SARIMAX prediction model had the lowest RMSE results, while the RNN-LSTM obtained the lowest MAPE, which suggest that any of these models can be used to predict new outbreaks with the LSTM having a slight advantage. In Table 1 a summary of the results of the performance metrics, is shown.

Table 2 shows the predicted cases for each year, with the values in parentheses representing the relative percentual error, calculated from ground truth. The models over-predicted cases in 2015 and 2016, and under-predicted cases for 2017. But in general, under-predicted the cases for the complete 2015–2017 period.

## 4. Discussion

Dengue virus was reintroduced in Panama in 1993, since then all four serotypes has circulating causing several outbreaks [7]. Panama is located in the tropical zone, where climatic factors are favorable to the proliferation of mosquitoes. Temperature have been described as a best predictor of the increase of Dengue cases. Pinto et al. [39], found that minimum and maximum temperature are strong weather predictors for the increase of dengue cases, whereas rainfall and relative humidity have little correlation with dengue cases, using a data from 2000 to 2007 from Singapore. Optimal temperatures for mosquitoes proliferation are 20 ${}^{\circ }$C to 35 ${}^{\circ }$C. Aedes aegypti mosquitoes will amplify and transmit dengue viruses only if exposed to temperatures withing the range of 20 ${}^{\circ }$C to 35 ${}^{\circ }$C [40].

In our study the distribution of Dengue incidences was accumulated in the intervals of 1–3 and 33–42 weeks, which correlate with a drop of temperature due to the raining season. Although, environmental temperature in Panama remains all the year between 24 ${}^{\circ }$C to 36 ${}^{\circ }$C, other factors as of relative humidity and the accumulated precipitation could influence in the increment of dengue incidence. Models to predict the dynamic of a vector disease based only environmental variables, has the challenge of the introduction and reintroduction of different dengue serotypes or genotype, during the study period, could also influence the increment of Dengue cases [7], as well as the introduction of other arboviruses like Zika in 2015 and Chikungunya in 2014, that are transmitted by same vector and cause a similar clinical disease [41,42]

The results of this study shows that climatic variables could be important factors to predict Dengue incidence behavior, especially those whom affected the Aedes infestation index. It was observed that incidence of Dengue cases increased around of 7–8 weeks after raining season begins, similar to what Vargas et al. [43], and Diaz et al. [44], reported previously.

The results of the correlations reflect the effect of a larger population of mosquitoes, it is due to a greater availability of places as breeding sites during the rainy season, which are also connected to urban regions with higher population density.

The correlations indicate the existence of a strong relationship between the climatic variables and the Dengue Incidence cases, with precipitation being an indicator that warns up to 8 weeks before the start of the Dengue case season, knowing the weeks where the highest average of cases are registered as well as an estimate of the total number of cases, the necessary supplies can be obtained for the different interventions that are carried out to preserve public health during Dengue epidemics, the predictive models will not tell us exactly when a case will occur and the exact quantity but they will provide lights when facing the unknown.

It is a public health priority to study diseases with the potential to affect an increasingly large population. In this spirit, this manuscript proposes traditional statistical time-series analysis (SARIMA and SARIMAX) and RNN-LSTM networks to study the effect of climate variables on the incidence of dengue cases in the Metropolitan region of Panama for the 1999–2017 period. In general both, SARIMAX and RNN-LSTM could be suggest as predictor of new outbreaks, with the LSTM having a slight advantage.

LSTM models have great potential due to their flexibility and the ability to learn from previous events of a series. In future work, these capabilities can be put to work to predict future outbreaks, special by novel Attention based RNN-LSTM models. They allow the network to automatically search for the values within the past values that are most influential for the prediction [45,46].

From the modeling results, it seems that although the initial correlations of Dengue incidence with climatic variables were statistical significant. These variables play a minor role in the effectiveness of the SARIMAX and RNN-LSTM models. This conclusions are not surpising as Dengue is a complex problem with many exogenous variables that are not controlled in models. For instance, some authors suggest that it can be attributed to the possibility of having almost constant temperatures throughout the year [47]. Also, there are other factors that can influence the dengue cases, such as: the distribution of objects unintentionally dispersed by man that can serve as a breeding ground once the water can recharge in them, collection of water in containers for human consumption due the limited access to drinking water, which is truly relevant in the case of the metropolitan area of Panama City [48]. A way to further understand this relation, would be to apply spatio-temporal analysis with climatic measurements for the area studied as presented by Nascimineto et al. [49], for brazilian dry climate regions. Also, a numerical analysis based on differential equations, in which compartments could be used to represent human-vector interactions, as well quantifying local vector erradication and control efforts [15,50,51], could help understand the transmission strategies beyond climatic variables.

Finally, Out-of-sample forecasting is more challenging as the changing nature of dengue in Panama over the past two decades does not guarantee that past incidence will be a an accurate predictor of future incidence. This underlines the crucial nature of long-term population and climate changes that will need to be incorporated into dengue forecasting models. In this regards a great deal of work has been advanced in the determination of climate at the end of the century for Panama, mostly focusing on precipitation. Many studies concur that precipitation will shift to earlier months of the year and that their intensity and duration (Consecutive Wet Days, CWD) will increase [52,53,54,55,56].

One of the limitations of this study, is that its scope was to only look at short-medium term predictions. Another limitation is the availability of the data, as some parts of the time series had to be imputed using known methodologies. Also, measurements from a single metoreological station is used to represent the climatic used in this study.

In recent years public health has become a priority, when facing the challenges that the future holds. For future work, it would be advantageous for Panama to use prediction techniques and the use of early warning strategies, would provide a clear path for mitigation of a future risk.

## Figures and Tables

**Table 1:** Summary of the performance metrics obtained by the 3 models studied.

Model	RMSE	MAPE
SARIMA (3,1,3)(1,1,1)(52)	25.83	112.70
SARIMAX (3,1,3)(1,1,1)(52)	25.76	108.44
LSTM 208 H.U.	26.16	59.68

**Table 2:** Summary of the prediction year-by-year of the 3 models studied.

Model	2015	2016	2017	Total
**Ground Truth**	**1478**	**1736**	**3005**	**6119**
SARIMA	1682 (14%)	1970 (20%)	1978 (−34%)	5631 (−8%)
SARIMAX	1558 (5%)	1882 (15%)	2001 (−33%)	5441 (−11%)
LSTM	1648 (11%)	1915 (17%)	1687 (−44%)	5241 (−14%)

## Data Availability

Incidence of Dengue cases data is of public domain, and are compiled yearly by MINSA and published in the Health Statistical Yearbook http://minsa.gob.pa/informacion-salud/anuarios-estadisticos, last accessed in 15 November 2021. Climate data are available at https://biogeodb.stri.si.edu/physical_monitoring/research/metpark, last accessed on 15 November 2021.

## Appendix A

**Table A1:** Analys of confirmed per-capita Dengue cases (cases per 1000 inhabitants) in rural and urban areas within the metropolitan region of Panama.

Year	Cases in Rural Areas	Cases in Urban Areas
2004	0.5	3.1
2005	5.9	15.2
2006	5.3	10.7
2007	4.0	6.3
2008	2.9	4.6
2009	1.7	18.9
2010	0.6	2.4
2011	10.6	9.0
2012	1.0	1.2
2013	3.6	11.8
2014	11.4	16.8
2015	3.2	7.8
2016	3.1	10.1
2017	4.2	12.9
Average	58	130.8
